# Rose Bengal and Riboflavin Mediated Photodynamic Antimicrobial Therapy Against Selected South Florida *Nocardia* Keratitis Isolates

**DOI:** 10.1167/tvst.11.1.29

**Published:** 2022-01-19

**Authors:** Ethan Adre, Heather Durkee, Alejandro Arboleda, Karam Alawa, Jorge Maestre, Keenan J. Mintz, Roger M. Leblanc, Guillermo Amescua, Jean–Marie Parel, Darlene Miller

**Affiliations:** 1 Ophthalmic Biophysics Center, Department of Ophthalmology, Bascom Palmer Eye Institute, University of Miami Miller School of Medicine, Miami, FL, USA; 2 Department of Biomedical Engineering, College of Engineering, University of Miami, Coral Gables, FL, USA; 3 Ocular Microbiology Laboratory, Department of Ophthalmology, Bascom Palmer Eye Institute, University of Miami Miller School of Medicine, Miami, FL, USA; 4 Department of Chemistry, College of Arts and Science, University of Miami, Coral Gables, FL, USA; 5 Anne Bates Leach Eye Hospital, Department of Ophthalmology, Bascom Palmer Eye Institute, University of Miami Miller School of Medicine, Miami, FL, USA

**Keywords:** photodynamic therapy, *Nocardia* keratitis, rose bengal, riboflavin, antibiotic resistance

## Abstract

**Purpose:**

To examine and compare the efficacy of in vitro growth inhibition using rose bengal and riboflavin photodynamic antimicrobial therapy (PDAT) for *Nocardia* keratitis isolates.

**Methods:**

*Nocardia asteroides* complex, *Nocardia amikacinitolerans*, and *Nocardia farcinica* species were isolated from patients with confirmed *Nocardia* keratitis. Isolates were tested against three experimental groups: (1) no photosensitizer/no irradiation, (2) photosensitizer/no irradiation, and (3) photosensitizer/irradiation. Each isolate was prepared in suspension to a concentration of 1.5 × 10^8^ CFU/mL. Bacterial suspensions were mixed with water or prepared 0.1% photosensitizer solution for a final bacterial concentration of 1.5 × 10^7^ CFU/mL. Aliquots of 1 mL were plated on 5% sheep blood agar. Rose bengal and riboflavin PDAT plates were irradiated for 15 minutes with a 525- or 375-nm custom 6-mW/cm^2^ powered light source for a total fluence of 5.4 J/cm^2^. All experimental groups were repeated in triplicate. Plates were incubated in a 35°C non-CO_2_ incubator for 96 hours and photographed. Percent inhibition was evaluated using LabVIEW-based software.

**Results:**

All strains of *Nocardia* tested with 0.1% rose bengal and irradiated for 15 minutes demonstrated statistically significant inhibition of growth (*P* < 0.05). No other experimental groups displayed any bacterial inhibition.

**Conclusions:**

Rose bengal is superior to riboflavin PDAT against selected *Nocardia* isolates*.* In vivo testing is warranted to investigate the utility of rose bengal PDAT for severe *Nocardia* keratitis.

**Translational Relevance:**

In vitro results for three clinical strains of *Nocardia* support the possible use of rose bengal PDAT as a complementary treatment of *Nocardia* keratitis*.*

## Introduction

*Nocardia* is an aerobic, Gram-positive, partially acid-fast, branching bacteria commonly found in dust, soil, water, and vegetative matter. Ocular *Nocardia* infections are rare, with a global prevalence below 2%.^1^ The majority of *Nocardia* keratitis cases are secondary to trauma,^1^^–^^5^ contact lens use,^6^^–^^12^ or iatrogenic^13^^–^^15^ causes. Given the diversity,^16^^–^^19^ regionality,^20^ rarity, and inexperience^21^ with this organism, it is often misdiagnosed.^3^^,^^13^^,^^22^^,^^23^ Initial management consists of multiple courses of topical, periocular, and systemic antimicrobials.^2^^,^^3^^,^^22^^,^^24^^,^^25^ *Nocardia* keratitis does not respond to typical broad-spectrum antibiotics,^7^^,^^25^^–^^32^ worsens with corticosteroid administration,^33^^,^^34^ and has demonstrated pocketed resistance to the standard-of-care treatments for *Nocardia*, including amikacin^1^^,^^6^^,^^10^^,^^24^^,^^35^ and trimethoprim-sulfamethoxazole.^30^^,^^31^^,^^36^ If not identified rapidly and treated properly, *Nocardia* keratitis can result in poor visual prognosis with patients having significant corneal scarring^11^^,^^37^ or corneal perforation^23^ with secondary severe intraocular complications.

In light of this, there is an unmet need for an effective treatment against *Nocardia* keratitis. Photodynamic antimicrobial therapy (PDAT) is a promising alternative treatment option. PDAT is a photochemical reaction that utilizes a photosensitive dye activated by a certain wavelength of light. When the dye is energized to an excited state, it interacts with ambient oxygen to generate reactive oxygen species capable of initiating cell death through a variety of mechanisms.^38^ An additional benefit of PDAT in corneal tissues is corneal strengthening via collagen crosslinking.^39^ Riboflavin emerged as the first photosensitizing agent used for corneal crosslinking in patients with keratoconus,^39^ and rose bengal has since been established as an effective photosensitizer for PDAT.^40^^–^^46^ Previous in vitro and in vivo studies have demonstrated the efficacy of PDAT against microbial keratitis of multiple etiologies.^41^^–^^43^^,^^47^^–^^49^ Further studies have successfully characterized the safety profile of rose bengal PDAT against microbial keratitis in humans, establishing its use as a viable therapy.^40^^,^^44^^–^^46^ This paper reports on and compares the in vitro responses of *Nocardia* corneal isolates against PDAT using two different photosensitizers: rose bengal and riboflavin.

## Materials and Methods

### Organism Selection

**Figure 1.**
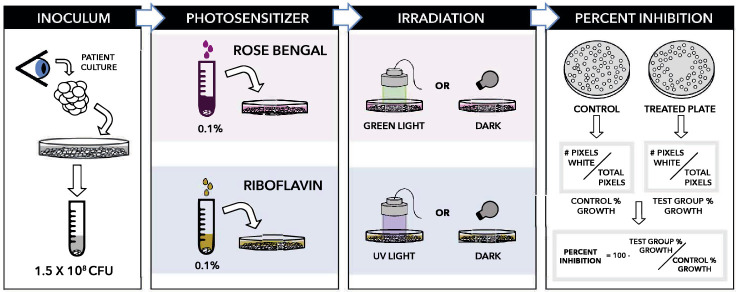
In vitro testing protocol of rose bengal and riboflavin PDAT. Culture-positive isolates are collected and prepared in suspension. Solutions are mixed with either 0.1% rose bengal or riboflavin. Plates are kept in the dark or irradiated with the appropriate light source. Photographs were taken and percent inhibition values were calculated after incubation. (Adapted from Halili et al.^43^)

Three isolates, *Nocardia asteroides* complex, *Nocardia amikacinitolerans* and *Nocardia farcinica* were chosen for experimentation. These species were among the most commonly isolated *Nocardia* species from Bascom Palmer Eye Institute. Each isolate was located in cold storage based on our ocular microbiology department records from patients with culture positive *Nocardia* recovered from the cornea. Prior to experimentation, each isolate was warmed to room temperature, subbed onto 5% sheep blood agar, and grown in a 35°C non-CO_2_ incubator for 72 hours to ensure maximal growth and reproductive viability and purity of the organisms (Fig. 1).

### Organism Characterization

In vitro antimicrobial susceptibilities of the *Nocardia* strains were determined through minimum inhibitory concentration breakpoints set by the Clinical and Laboratory Standards Institute.^50^ Isolates were referred to an outside laboratory (Focus Diagnostics Infectious Disease, Cypress, CA) for identification and susceptibility testing. Intermediate susceptibilities were considered resistant to the accompanying antibiotic. It is important to note that there are no antibiotic susceptibility breakpoints for topical antibiotic therapy.

### Photosensitizer Preparation

Photosensitizing solutions were prepared the day of experimentation and kept in light-protected tubes to prevent photobleaching. Photosensitizing solutions of 0.1% concentration were prepared by mixing a ratio of 1 mg powder to 1 mL sterile water for rose bengal (330000; Sigma-Aldrich, St. Louis, MO) and riboflavin (R7774; Sigma-Aldrich). Photosensitizer concentrations were evaluated post hoc with UV-visible spectroscopy.

### Organism Preparation for PDAT

Each isolate was grown in 5% sheep blood agar plates for 72 hours before experimentation. Colonies were suspended in sterile water and adjusted to a 0.5 McFarland (1.5 × 10^8^ CFU/mL) solution (Fig. 1).

### PDAT

To test the in vitro response of the microorganisms to PDAT, three experimental groups were set for each photosensitizer: (1) growth control (no irradiation, no photosensitizer); (2) rose bengal or riboflavin dark (photosensitizer, no irradiation); and (3) PDAT (photosensitizer with irradiation). Groups were repeated in triplicate with both rose bengal and riboflavin. All experiments were conducted in minimal residual lighting of <1 lux, confirmed with a digital lux meter (LX1010B; Sinometer Instruments, Shenzhen, China).

**Figure 2.**
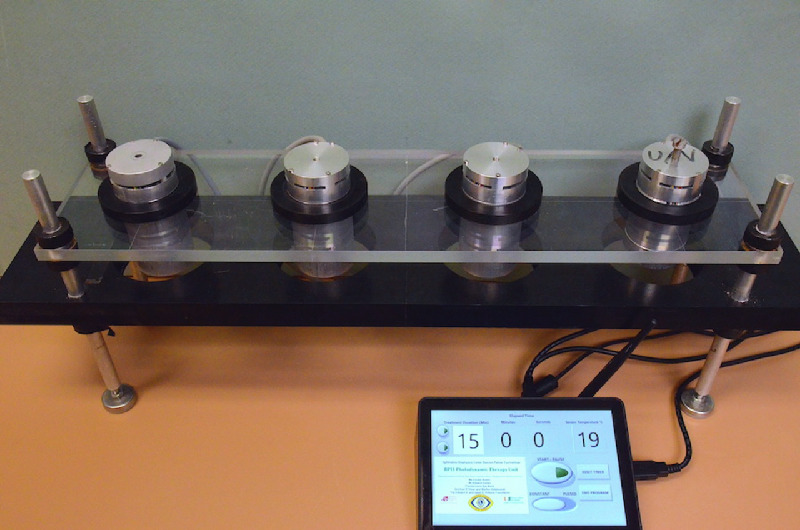
Custom-made in vitro PDAT testing station. This setup allows for simultaneous testing of three sample plates with 525-nm light and an additional single 375-nm lamp on the right. Each light source is computer controlled with custom LabVIEW software.

Isolates were then diluted to a final working concentration of 1.5 × 10^7^ CFU/mL with either sterile water or photosensitizer solution. Aliquots of 1 mL of the growth control, rose bengal, or riboflavin solutions were then pipetted to individual 5% sheep blood agar plates, evenly distributed on the surface, and decanted of the excess solution. Plates in group 3 were irradiated for 15 minutes with either 525-nm light for rose bengal or 375-nm light for riboflavin. The light sources (Fig. 2) were developed in-house with 6-mW/cm^2^ power density measured with a power meter (PM200; Thorlabs, Newton, NJ) and sensor (S130C; Thorlabs). The 525- and 375-nm irradiation sources produced a uniform irradiance of 6 mW/cm^2^ over a circular surface of 47 mm diameter, and the plates were exposed for 15 minutes, producing a fluence of 5.4 J/cm^2^.^41^^–^^43^ Previous testing showed that, in this setup, there is no increase in heat during irradiation.^42^

After irradiation, all plates were secured and shielded from light with aluminum foil and incubated at 35°C in a non-CO_2_ incubator for 96 hours before observation. Photographs of each plate were taken after 96 hours to evaluate percent inhibition.

Percent growth inhibition on the agar plates was calculated using a custom software in LabVIEW, described previously.^41^^–^^43^ Briefly, the percent growth was calculated from a grayscale image:

$$\begin{array}{c} \mathrm{Percent}\;\mathrm{growth}=\frac{\#\;\mathrm{White}\;\mathrm{pixels}}{\mathrm{Total}\;\#\;\mathrm{pixels}\;\mathrm{in}\;\mathrm{central}\;\mathrm{zone}}, \end{array}$$

with control group (1) considered 100% growth. Percent inhibition was then calculated:

$$\begin{array}{c} \mathrm{Percent}\;\mathrm{inhibition}=100\%-\frac{\mathrm{Experiemental}\;\mathrm{group}\;\mathrm{percent}\;\mathrm{growth}}{\mathrm{Growth}\;\mathrm{control}\;\mathrm{percent}\;\mathrm{growth}}. \end{array}$$

ANOVA testing was used to assess the percent inhibition between experimental groups with significance set at *P* < 0.05.

## Results

### Nocardia Isolate Susceptibility Profiles

Antimicrobial susceptibility profiles are described in the Table. *Nocardia asteroides* complex was resistant to 8 of the 13 antibiotics tested. *Nocardia amikacinitolerans* and *Nocardia farcinica* were resistant to 7 of the 13 antibiotics tested. All organisms were resistant to cefepime, ciprofloxacin, clarithromycin, and doxycycline.

**Table.** Nocardia Isolate Antibiotic Susceptibility Profiles

Antibiotic Class	Antibiotic	*Nocardia asteroides* Complex	*Nocardia amikacinitolerans*	*Nocardia farcinica*
Aminoglycoside	Amikacin	R	R	S
	Tobramycin	S	S	R
Penicillin/cephalosporin	Amoxicillin/clavulanic	S	S	S
	Ceftriaxone	R	S	R
	Cefepime	R	R	R
Fluoroquinolone	Ciprofloxacin	R	R	R
	Moxifloxacin	R	R	S
Macrolide	Clarithromycin	R	R	R
Tetracycline	Doxycycline	R	R	R
	Minocycline	S	S	R
Carbapenem	Imipenem	R	R	S
Oxazolidinone	Linezolid	S	S	S
Sulfonamide	Trimethoprim/sulfamethoxazole	S	S	S

R, resistant; S, susceptible.

### Photosensitizing Solutions

Spectroscopy of each photosensitizer solution measured extinction coefficients for rose bengal (85,410 L mol^−1^ cm^−1^) and riboflavin (8140 L mol^−1^ cm^−1^) in order to determine the concentrations. Final concentrations of rose bengal and riboflavin solutions were calculated to be 0.12% ± 0.002% and 0.09% ± 0.006%, respectively.

### PDAT

**Figure 3.**
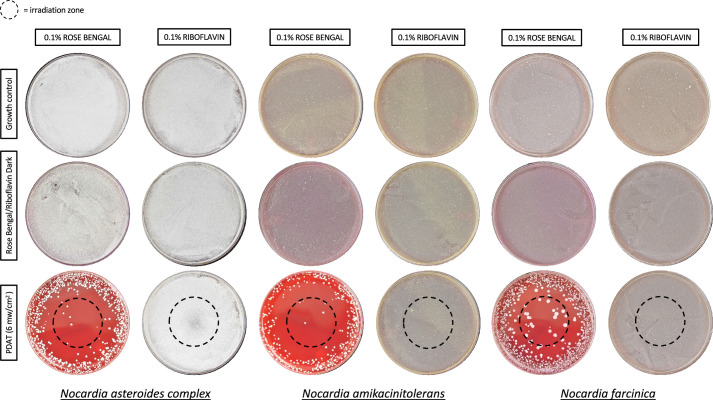
*Nocardia* growth after incubation. Representative photographs of each experimental group for three *Nocardia* isolates taken 96 hours after experimentation. The irradiation zone represents the central 47-mm area that corresponds to the diameter of the PDAT lamp.

**Figure 4.**
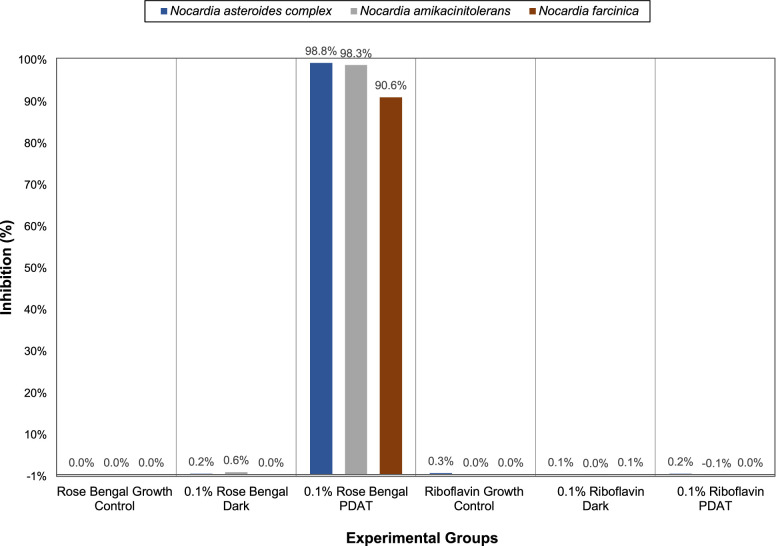
Percent inhibition of *Nocardia* keratitis isolates growth in response to treatment. The values for each *bar* represent the average percent inhibition of the three trials for each experimental group.

Photographs of the plates taken at 96 hours are shown in Figure 3. Group 3 (0.1% rose bengal PDAT with 525 nm irradiation) showed 98.8%, 98.3%, and 90.6% inhibition against *Nocardia asteroides* complex, *Nocardia amikacinitolerans*, and *Nocardia farcinica*, respectively. Comparatively, in group 3, riboflavin PDAT with 375-nm irradiation demonstrated less than 0.2% in all tested *Nocardia* strains. In group 2, rose bengal alone and riboflavin alone demonstrated less than 1% inhibition for all *Nocardia* strain. Percent inhibition for all tested strains and experimental groups are shown in Figure 4.

### Statistical Analysis

A one-way ANOVA test determined there was a statistically significant difference among all groups for each species: *Nocardia asteroides* complex*,* F(5,12) = 1638, *P* < 0.001; *Nocardia amikacinitolerans*, *F*(5,12) = 8276, *P* < 0.001; and *Nocardia farcinica*, *F*(5,12) = 31806, *P* < 0.001. A Tukey honest significance difference test was conducted post hoc to compare the significance between individual groups. The experimental groups could be divided into two tiers of inhibition, all or none, with no significant overlap. The presence of 0.1% rose bengal with 525-nm irradiation demonstrated the only significant percent inhibition when compared with all other experimental groups tested.

## Discussion

This is the first study to compare in the in-vitro responses of *Nocardia* keratitis isolates to rose bengal and riboflavin PDAT. We found that 0.1% rose bengal PDAT significantly inhibits the growth of *Nocardia asteroides* complex, *Nocardia amikacinitolerans*, and *Nocardia farcinica* isolates. No other experimental groups demonstrated growth inhibition.

This study further supports the use of rose bengal as a photosensitizer for PDAT due to its broad efficacy against multiple bacterial and fungal species. Other studies have found similar results for the in vitro inhibition of PDAT against other microbial keratitis isolates, including *Fusarium*, *Aspergillus*, *Candida*,^41^ *Staphylococcus*,^43^ and *Pseudomonas*^42^ species. These studies also demonstrated significant inhibition using rose bengal as a photosensitizer and limited success using riboflavin. The superior efficacy of rose bengal over riboflavin may be attributed to the greater singlet oxygen yield of rose bengal.^51^ Singlet oxygen production of rose bengal may be greater due in part to the increased ability of rose bengal to absorb light. Our measured extinction coefficient of rose bengal (85,410 L mol^−1^ cm^−1^) was 10 times greater than that for riboflavin (8140 L mol^−1^ cm^−1^), demonstrating that rose bengal was a better absorber of light and offers the potential for increased production of singlet oxygen. Also, it is well known that, when activated with light, rose bengal exclusively produces singlet oxygen (type 2 mechanism) in systems without a donor to facilitate the formation of the superoxide anion, whereas riboflavin produces both singlet oxygen and other reactive oxygen species (ROS) through electron transfer (type 1 mechanism).^51^ This difference in ROS production may contribute to the increased effect seen from rose bengal because more singlet oxygen is produced through this photosensitizer.^52^ A final potential difference between the efficacies of rose bengal and riboflavin PDAT is that the 375-nm light used to activate the riboflavin is competitively absorbed by bacteria^53^; therefore, less light is available to activate the riboflavin to create ROS and singlet oxygen.

Furthermore, it has been established that *Nocardia* species carry genes coding for catalase^54^ and superoxide dismutase,^55^^,^^56^ which allow them to neutralize free radicals. In vitro experiments have found that *Nocardia* species are able to survive oxidative metabolic bursts from neutrophils through direct activity of these two enzymes.^54^^–^^56^ If catalase and superoxide dismutase demonstrate greater affinity for neutralizing non-singlet ROS, which are more readily produced with activation of riboflavin,^57^ it is conceivable that this would reduce the antimicrobial efficacy of riboflavin PDAT. More studies should be performed to find if these defense mechanisms have an effect and to evaluate for any strain-specific resistance to PDAT. The inherent free-radical scavenging system of *Nocardia* may play a role in the organism's resistance to riboflavin PDAT.

In this set of *Nocardia* isolates, we found a high resistance to empiric, broad-spectrum antibiotics, including ciprofloxacin and moxifloxacin, in addition to resistance to first-line^35^ amikacin treatment in two of the three isolates in this study. Previous studies have characterized antibiotic resistance patterns for *Nocardia*. The reported percent resistance for ciprofloxacin ranged between 45% and 83%,^19^^,^^28^^,^^30^^,^^58^^,^^59^ moxifloxacin was 60%,^19^^,^^28^ and amikacin was between 0% and 11.8%.^19^^,^^28^^,^^30^^,^^58^^,^^59^ Our results align with the general profile of fluoroquinolone resistance but differ with more prominent amikacin resistance. These differences between our susceptibility results and those previously described can be due to a combination of the regionality^32^ of *Nocardia* species and strain-specific characteristics.^16^^,^^20^ Case reports have also noted resistance to amikacin in multiple *Nocardia* species,^4^^,^^7^^,^^10^^,^^25^^,^^29^ including *Nocardia asteroides* complex and *Nocardia amikacinitolerans*, as examined in our study. For clinicians, this highlights the importance of species identification and susceptibility testing to cater treatment regimen. A single, blanket antibiotic therapy is not effective in treating *Nocardia* keratitis.

Overall, this study provides the basis for further experimentation and characterization of *Nocardia* keratitis and its broader response to PDAT. With trends continuing, we will likely discover more antibiotic resistance among other *Nocardia* species and establish greater reason to incorporate alternative antimicrobial strategies such as rose bengal PDAT. The success of this study warrants further examination of the in vivo response of *Nocardia* to rose bengal PDAT. Rose bengal PDAT may even be considered in cases of severe, refractory *Nocardia* keratitis as a last resort before surgical intervention.

## References

[1] DeCroos FC, Garg P, Reddy AK, et al. Optimizing diagnosis and management of Nocardia keratitis, scleritis, and endophthalmitis: 11-year microbial and clinical overview. *Ophthalmology*. 2011; 118: 1193–1200. 21276615 10.1016/j.ophtha.2010.10.037

[2] Trichet E, Cohen-Bacrie S, Conrath J, Drancourt M, Hoffart L. Nocardia transvalensis keratitis: an emerging pathology among travelers returning from Asia. *BMC Infect Dis*. 2011; 11: 296–298. 22040176 10.1186/1471-2334-11-296 PMC3234200

[3] Nor-Sharina Y, Lee KF, Siti Hawa H, Zunaina E. Nocardia keratitis presenting as viral disciform keratitis, a case report. *Int J Ophthalmol*. 2011; 11: 8–10.

[4] Pandya VB, Petsoglou C. Nocardia transvalensis resistant to amikacin: an unusual cause of microbial keratitis. *Cornea*. 2008; 27: 1082–1085. 18812779 10.1097/ICO.0b013e3181783a20

[5] Sheemar S, Chand K, Kaur J, Moudgill T. Nocardia keratitis: a case report from tertiary care hospital of Punjab, India. *J Med Soc*. 2017; 31: 211–213.

[6] Patel R, Sise A, Al-Mohtaseb Z, et al. Nocardia asteroides keratitis resistant to amikacin. *Cornea*. 2015; 34: 1617–1619. 26418432 10.1097/ICO.0000000000000634

[7] Eggink CA, Wesseling P, Boiron P, Meis JF. Severe keratitis due to Nocardia farcinica. *J Clin Microbiol*. 1997; 35: 999–1001. 9157173 10.1128/jcm.35.4.999-1001.1997 PMC229721

[8] Sharma N, O'Hagan S. The role of oral co-trimoxazole in treating Nocardia farcinica keratitis: a case report. *J Ophthalmic Inflamm Infect*. 2016; 6: 21–25. 27294730 10.1186/s12348-016-0087-y PMC4905934

[9] Gieger A, Waller S, Pasternak J. Nocardia arthritidis keratitis: case report and review of the literature. *Nepal J Ophthalmol*. 2017; 9: 91–94. 29022964 10.3126/nepjoph.v9i1.17543

[10] Johansson B, Fagerholm P, Petranyi G, Claesson Armitage M, Lagali N. Diagnostic and therapeutic challenges in a case of amikacin-resistant Nocardia keratitis. *Acta Ophthalmol*. 2017; 95: 103–105. 27572657 10.1111/aos.13182

[11] Shah P, Zhu D, Culbertson WW. Therapeutic femtosecond laser-assisted lamellar keratectomy for multidrug-resistant Nocardia keratitis. *Cornea*. 2017; 36: 1429–1431. 28834821 10.1097/ICO.0000000000001318

[12] Behaegel J, Ni Dhubhghaill S, Koppen C. Diagnostic challenges in Nocardia keratitis. *Eye Contact Lens*. 2018; 44: S370–S372. 10.1097/ICL.0000000000000462 29219900

[13] Bhusal B, Kumar A, Prajna MV, Srinivasan M. Nocardia keratitis following penetrating corneal injury treated with topical ampicillin. *Nepal J Ophthalmol*. 2016; 8: 82–86. 28242891 10.3126/nepjoph.v8i1.16143

[14] Javadi MA, Kanavi MR, Zarei-Ghanavati S, et al. Outbreak of Nocardia keratitis after photorefractive keratectomy: clinical, microbiological, histopathological, and confocal scan study. *J Cataract Refract Surg*. 2009; 35: 393–398. 19185261 10.1016/j.jcrs.2008.08.045

[15] Prieto-Borja L, Garcia-Coca M, Ustratova I, Alejandre Alba N. Keratitis due to Nocardia nova after cataract surgery. *Enferm Infecc Microbiol Clin*. 2017; 35: 57–58. 27448807 10.1016/j.eimc.2016.06.004

[16] Conville PS, Brown-Elliott BA, Smith T, Zelazny AM. The complexities of Nocardia taxonomy and identification. *J Clin Microbiol*. 2018; 56: e01419–01417. 29118169 10.1128/JCM.01419-17 PMC5744224

[17] Hashemi-Shahraki A, Heidarieh P, Bostanabad SZ, et al. Genetic diversity and antimicrobial susceptibility of Nocardia species among patients with nocardiosis. *Sci Rep*. 2015; 5: 17862. 26638771 10.1038/srep17862 PMC4671095

[18] Rodriguez-Nava V, Couble A, Devulder G, Flandrois JP, Boiron P, Laurent F. Use of PCR-restriction enzyme pattern analysis and sequencing database for hsp65 gene-based identification of Nocardia species. *J Clin Microbiol*. 2006; 44: 536–546. 16455910 10.1128/JCM.44.2.536-546.2006 PMC1392680

[19] Schlaberg R, Fisher MA, Hanson KE. Susceptibility profiles of Nocardia isolates based on current taxonomy. *Antimicrob Agents Chemother*. 2014; 58: 795–800. 24247124 10.1128/AAC.01531-13 PMC3910866

[20] McTaggart LR, Richardson SE, Witkowska M, Zhang SX. Phylogeny and identification of Nocardia species on the basis of multilocus sequence analysis. *J Clin Microbiol*. 2010; 48: 4525–4533. 20844218 10.1128/JCM.00883-10 PMC3008441

[21] Mascarenhas J, Srinivasan M, Chen M, et al. Differentiation of etiologic agents of bacterial keratitis from presentation characteristics. *Int Ophthalmol*. 2012; 32: 531–538. 22752605 10.1007/s10792-012-9601-x PMC3603562

[22] Bajracharya L, Gurung R. A case of Nocardia keratitis treated successfully with topical amikacin. *Nepal J Ophthalmol*. 2012; 4: 170–173. 22344016 10.3126/nepjoph.v4i1.5870

[23] Seyyed-Yousefi SZ, Fatahi-Bafghi M, Eshraghi SS, et al. Observation of Nocardia asteroides complex in a patient with corneal ulceration: the first case report from Iran. *J Kerman Univ Med Sci*. 2016; 23: 228–233.

[24] Rahimi F, Aghsaie Fard M, Soltani Mogaddam R. A case of amniotic membrane transplantation in non-healing Nocardia asteroides keratitis. *J Ocul Biol Dis Infor*. 2009; 2: 37–39. 20072646 10.1007/s12177-009-9019-5 PMC2802505

[25] Johansson B, Fagerholm P, Armitage MC, Stenevi U, Petranyi G, Lagali N. Case report: bacterial keratitis with an unusual and elusive etiologi: Nocardia beijingensis. *Acta Ophthalmol*. 2014; 92: 14.

[26] Kogure T, Shimada R, Ishikawa J, et al. Homozygous triplicate mutations in three 16S rRNA genes responsible for high-level aminoglycoside resistance in Nocardia farcinica clinical isolates from a Canada-wide bovine mastitis epizootic. *Antimicrob Agents Chemother*. 2010; 54: 2385–2390. 20308368 10.1128/AAC.00021-10 PMC2876378

[27] Komaki H, Ichikawa N, Hosoyama A, et al. Genome based analysis of type-I polyketide synthase and nonribosomal peptide synthetase gene clusters in seven strains of five representative Nocardia species. *BMC Genomics*. 2014; 15: 323. 24884595 10.1186/1471-2164-15-323 PMC4035055

[28] McTaggart LR, Doucet J, Witkowska M, Richardson SE. Antimicrobial susceptibility among clinical Nocardia species identified by multilocus sequence analysis. *Antimicrob Agents Chemother*. 2015; 59: 269–275. 25348540 10.1128/AAC.02770-14 PMC4291361

[29] Wilson RW, Steingrube VA, Brown BA, et al. Recognition of a Nocardia transvalensis complex by resistance to aminoglycosides, including amikacin, and PCR-restriction fragment length polymorphism analysis. *J Clin Microbiol*. 1997; 35: 2235–2242. 9276394 10.1128/jcm.35.9.2235-2242.1997 PMC229946

[30] Valdezate S, Garrido N, Carrasco G, Villalón P, Medina-Pascual MJ, Saéz-Nieto JA. Resistance gene pool to co-trimoxazole in non-susceptible Nocardia strains. *Front Microbiol*. 2015; 6: 376. 25972856 10.3389/fmicb.2015.00376 PMC4412068

[31] Mehta H, Weng J, Prater A, Elworth RAL, Han X, Shamoo Y. Pathogenic Nocardia cyriacigeorgica and Nocardia nova evolve to resist trimethoprim-sulfamethoxazole by both expected and unexpected pathways. *Antimicrob Agents Chemother*. 2018; 62: e00364–e00418. 29686152 10.1128/AAC.00364-18 PMC6021631

[32] Zhao P, Zhang X, Du P, Li G, Li L, Li Z. Susceptibility profiles of Nocardia spp. to antimicrobial and antituberculotic agents detected by a microplate Alamar Blue assay. *Sci Rep*. 2017; 7: 43660. 28252662 10.1038/srep43660 PMC5333629

[33] Srinivasan M, Mascarenhas J, Rajaraman R, et al. Corticosteroids for bacterial keratitis: the Steroids for Corneal Ulcers Trial (SCUT). *Arch Ophthalmol*. 2012; 130: 143–150. 21987582 10.1001/archophthalmol.2011.315 PMC3830549

[34] Srinivasan M, Mascarenhas J, Rajaraman R, et al. The Steroids for Corneal Ulcers Trial (SCUT): secondary 12-month clinical outcomes of a randomized controlled trial. *Am J Ophthalmol*. 2014; 157: 327–333.e3. 24315294 10.1016/j.ajo.2013.09.025 PMC3946996

[35] Lalitha P . Nocardia keratitis. *Curr Opin Ophthalmol*. 2009; 20: 318–323. 19387343 10.1097/ICU.0b013e32832c3bcc

[36] Mittal V, Fernandes M. Cotrimoxazole-resistant Nocardia sclerokeratitis: effective therapy with fourth-generation fluoroquinolones. *Can J Ophthalmol*. 2012; 47: e58–e60. 23217523 10.1016/j.jcjo.2012.07.007

[37] Sharma D, Mathur U, Gour A, Acharya M, Gupta N, Sapra N. Nocardia infection following intraocular surgery: report of seven cases from a tertiary eye hospital. *Indian J Ophthalmol*. 2017; 65: 371–375. 28573992 10.4103/ijo.IJO_564_16 PMC5565894

[38] Hamblin MR, Hasan T. Photodynamic therapy: a new antimicrobial approach to infectious disease? *Photochem Photobiol Sci*. 2004; 3: 436–450. 15122361 10.1039/b311900a PMC3071049

[39] Wollensak G, Spoerl E, Seiler T. Riboflavin/ultraviolet-a-induced collagen crosslinking for the treatment of keratoconus. *Am J Ophthalmol*. 2003; 135: 620–627. 12719068 10.1016/s0002-9394(02)02220-1

[40] Amescua G, Arboleda A, Nikpoor N, et al. Rose rengal photodynamic antimicrobial therapy: a novel treatment for resistant Fusarium keratitis. *Cornea*. 2017; 36: 1141–1144. 28691942 10.1097/ICO.0000000000001265 PMC5546001

[41] Arboleda A, Miller D, Cabot F, et al. Assessment of rose bengal versus riboflavin photodynamic therapy for inhibition of fungal keratitis isolates. *Am J Ophthalmol*. 2014; 158: 64–70. 24792103 10.1016/j.ajo.2014.04.007 PMC4075940

[42] Durkee H, Arboleda A, Aguilar MC, et al. Rose bengal photodynamic antimicrobial therapy to inhibit Pseudomonas aeruginosa keratitis isolates. *Lasers Med Sci*. 2019; 35: 861–866. 31872325 10.1007/s10103-019-02871-9 PMC7261617

[43] Halili F, Arboleda A, Durkee H, et al. Rose bengal- and riboflavin-mediated photodynamic therapy to inhibit methicillin-resistant Staphylococcus aureus keratitis isolates. *Am J Ophthalmol*. 2016; 166: 194–202. 27016125 10.1016/j.ajo.2016.03.014

[44] Martinez JD, Arboleda A, Naranjo A, et al. Long-term outcomes of riboflavin photodynamic antimicrobial therapy as a treatment for infectious keratitis. *Am J Ophthalmol Case Rep*. 2019; 15: 100481. 31198886 10.1016/j.ajoc.2019.100481 PMC6556526

[45] Martinez JD, Naranjo A, Amescua G, et al. Human corneal changes after rose bengal photodynamic antimicrobial therapy for treatment of fungal keratitis. *Cornea*. 2018; 37: e46–e48. 30028750 10.1097/ICO.0000000000001701 PMC6131034

[46] Naranjo A, Arboleda A, Martinez JD, et al. Rose bengal photodynamic antimicrobial therapy for patients with progressive infectious keratitis: a pilot clinical study. *Am J Ophthalmol*. 2019; 208: 387–396. 31493402 10.1016/j.ajo.2019.08.027 PMC7184264

[47] Schrier A, Greebel G, Attia H, Trokel S, Smith EF. In vitro antimicrobial efficacy of riboflavin and ultraviolet light on Staphylococcus aureus, methicillin-resistant Staphylococcus aureus, and Pseudomonas aeruginosa. *J Refract Surg*. 2009; 25: S799–S802. 19772254 10.3928/1081597X-20090813-07

[48] Martins SA, Combs JC, Noguera G, et al. Antimicrobial efficacy of riboflavin/UVA combination (365 nm) in vitro for bacterial and fungal isolates: a potential new treatment for infectious keratitis. *Invest Ophthalmol Vis Sci*. 2008; 49: 3402–3408. 18408193 10.1167/iovs.07-1592

[49] Naranjo A, Pelaez D, Arrieta E, et al. Cellular and molecular assessment of rose bengal photodynamic antimicrobial therapy on keratocytes, corneal endothelium and limbal stem cell niche. *Exp Eye Res*. 2019; 188: 107808. 31539544 10.1016/j.exer.2019.107808

[50] Woods GL, Wengenack NL, Lin G, et al. *Susceptibility Testing of Mycobacteria, Nocardia spp., and Other Aerobic Actinomyecetes*. Wayne, PA: Clinical and Laboratory Standards Institute; 2018. 31339680

[51] Redmond RW, Kochevar IE. Medical applications of rose bengal- and riboflavin-photosensitized protein crosslinking. *Photochem Photobiol*. 2019; 95: 1097–1115. 31111489 10.1111/php.13126

[52] Redmond RW, Gamlin JN. A compilation of singlet oxygen yields from biologically relevant molecules. *Photochem Photobiol*. 1999; 70: 391–475. 10546544

[53] Lefta SH, Madlum KN, Aldayem WA, Abdallah KM. Antibacterial effect of silver nanoparticles on Pseudomonas aeruginosa bacteria. *Int J ChemTech Res*. 2016; 9: 382–390.

[54] Filice GA. Resistance of Nocardia asteroides to oxygen-dependent killing by neutrophils. *J Infect Dis*. 1983; 148: 861–867. 6355320 10.1093/infdis/148.5.861

[55] Beaman BL, Black CM, Doughty F, Beaman L. Role of superoxide dismutase and catalase as determinants of pathogenicity of Nocardia asteroides: importance in resistance to microbicidal activities of human polymorphonuclear neutrophils. *Infect Immun*. 1985; 47: 135–141. 3880721 10.1128/iai.47.1.135-141.1985 PMC261488

[56] Revol A, Espinoza-Ruiz M, Medina-Villanueva I, Salinas-Carmona MC. Expression of Nocardia brasiliensis superoxide dismutase during the early infection of murine peritoneal macrophages. *Can J Microbiol*. 2006; 52: 1255–1260. 17473895 10.1139/w06-075

[57] Kamaev P, Friedman MD, Sherr E, Muller D. Photochemical kinetics of corneal cross-linking with riboflavin. *Invest Ophthalmol Vis Sci*. 2012; 53: 2360–2367. 22427580 10.1167/iovs.11-9385

[58] Reddy AK, Garg P, Kaur I. Speciation and susceptibility of Nocardia isolated from ocular infections. *Clin Microbiol Infect*. 2010; 16: 1168–1171. 19832708 10.1111/j.1469-0691.2009.03079.x

[59] Lalitha P, Tiwari M, Prajna NV, Gilpin C, Prakash K, Srinivasan M. Nocardia keratitis: species, drug sensitivities, and clinical correlation. *Cornea*. 2007; 26: 255–259. 17413948 10.1097/ICO.0b013e318033d853

